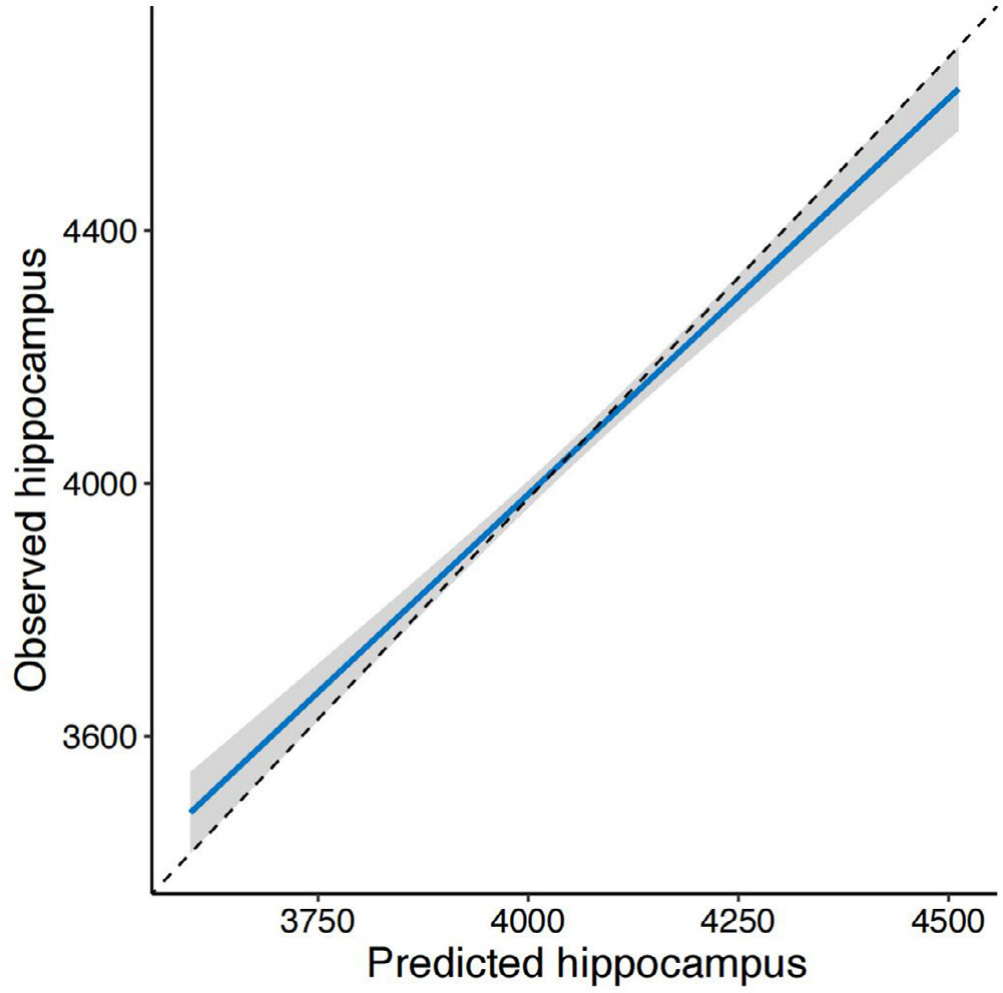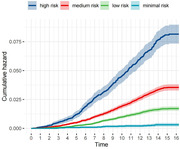# Proteomics‐Based Prediction of Hippocampal Atrophy in the General Population

**DOI:** 10.1002/alz.090326

**Published:** 2025-01-09

**Authors:** Meilin Chen, Zhibo Wang, Yuye Ning, Jianping Jia

**Affiliations:** ^1^ Xuanwu Hospital, Capital Medical University, Beijing, Beijing China; ^2^ Beijing Key Laboratory of Geriatric Cognitive Disorders, Beijing China; ^3^ Key Laboratory of Neurodegenerative Diseases, Ministry of Education, Beijing China; ^4^ Center of Alzheimer’s Disease, Beijing Institute for Brain Disorders, Beijing China; ^5^ Innovation Center for Neurological Disorders, Xuanwu Hospital, Capital Medical University, Beijing, Beijing China; ^6^ Innovation Center for Neurological Disorders, Xuanwu Hospital, Capital Medical University, Beijing, China;, Beijing China

## Abstract

**Background:**

Hippocampal atrophy is being characterized as a key biomarker in neurodegenerative disease, especially for dementia. Nevertheless, detecting hippocampus relied on magnetic resonance imaging (MRI), it's very costly and time‐consuming. Our objective is to develop a predictive score to detect MRI‐related hippocampal volume based on large‐scale blood proteomics.

**Method:**

This prospective cohort study utilized data from the UK Biobank, encompassing baseline assessments from 2006 to 2010 and follow‐up data up to September 2023. We focused on 5,680 participants with available Olink blood proteomics data (approximately 3000 proteins) and MRI‐related hippocampal volume. Feature selection and developing predictive score were used a least absolute shrinkage and selection operator (LASSO) regression. Moreover, we tested the prognostic value of the predictive model in the prediction of dementia outcomes in participants without known dementia with over 10‐years follow‐up period.

**Result:**

Our predictive score, incorporating 74 distinct proteins, was trained in a cohort of 4,544 individuals and validated in the additional cohort of 1,136 individuals. The predictive score accurately predicted MRI‐related hippocampal volume in the validation cohorts (Figure 1). Based on the predictive scores, we created four groups: high risk, medium risk, low risk, and minimal risk, according to selected cutoff values (10%, 50%, and 90%). The score was effective in identifying individuals at risk of dementia risk, thereby allowing stratification to different risk groups for dementia incidence. The hazard ratio for dementia incidence in the minimal risk group was 0.04 [95%CI: 0.02‐0.06] compared with the high‐risk group (Figure 2). Moreover, the predictive score was still effective in identifying individuals at risk of dementia risk in the independently cohort of 45,000 participants.

**Conclusion:**

The predictive scores, based on simple blood proteins, accurately predicts hippocampal volume and assesses the risk of dementia incidence. The score may enhance early screening for hippocampal atrophy and provide guidance for preventive care in early dementia.